# The complete chloroplast genome sequence of *Bryophyllum daigremontianum* (Crassulaceae)

**DOI:** 10.1080/23802359.2020.1867015

**Published:** 2021-02-08

**Authors:** Xiaojiao Zhou, Likuan Liu, Lin Xu, Kexin Song, Yuhua Shi, Tuansheng Shi, Xiangyu Tian

**Affiliations:** a School of Life Sciences, Zhengzhou University, Zhengzhou, Henan, P. R. China; b Lab of Environment and Resource in Qinghai-Tibet Plateau, Ministry of Education, Qinghai Normal University, Xining, Qinghai, P. R. China; c College of Surveying and Mapping Engineering, Yellow River Conservancy Technical Institute, Kaifeng, Henan, P. R. China

**Keywords:** *Bryophyllum daigremontianum*, complete chloroplast genome, phylogenetic studies

## Abstract

*Bryophyllum daigremontianum* is a very important traditional medicine and ornamental plant. Although *Bryophyllum* and *Kalanchoe* have been supported to form a clade, however, lack of chloroplast genomic severely hinders our understanding the phylogenetic relationships between them. In this study, the complete chloroplast genome of *B. daigremontianum* is first presented. It is 150,058 bp in length consisted a large single-copy (LSC, 82,164 bp) and a small single-copy (SSC, 17,042bp) separated by a pair of inverted repeats (IR, 25,426 bp) including 86 protein-coding genes, 37 tRNA, and 8 rRNA. Phylogenetic analysis supported that *B. daigremontianum* was closer to *K. tomentosa* than other species, which showed that chloroplast genome sequences offer a useful resource for future phylogenetic studies of *Kalanchoe* and *Bryophyllum* species.

*Bryophyllum daigremontianum* (Raym.-Hamet & Perrier) A. Berger is well known for its interesting reproduction and important traditional medicine distributed in tropics, such as South Asia, South Africa, and Australia (Bernard 2006; Milad et al. 2014; Kolodziejczyk-Czepas and Stochmal 2017; Stefanowicz-Hajduk et al. 2020). It is closely related to the genus *Kalanchoe* based on the morphology and molecular phylogenetic analysis, such as *matK*, *ITS* (Mort et al. 2001, 2005). Previous studies also found the species could hybrids between *Bryophyllum* Berger and *Kalanchoe* Adans., which makes the boundary of species between these two genera more confused (Hart and Eggli 1996; Van Ham and Hart 1998).

In recent years, chloroplast genomes have important contributions to phylogenetic studies of plants (Daniell et al. 2016). However, few chloroplast genomic data are available for these genera, only one species of *Kalanchoe* in GenBank database, *K. tomentosa* Baker (Accession number: MN794319). As part of the phylogenetic study to understand the relationship between *Bryophyllum* and *Kalanchoe*, we reported the whole chloroplast genome of *B. daigremontianum* and its phylogenetic analysis with related species.

Sample of *B. daigremontianum* was collected from Zhengzhou, Henan province, China (34°76′N, 119°72′E), specimen in the herbarium of Zhengzhou University (no. ZZU2020-201). High-quality genomic DNA was extracted from fresh leaves using Tiangen Plant Genomic DNA Kits (TIANGEN, Beijing) and directly constructed short-insert of 150 bp in length libraries and sequenced on the Illumina Genome Analyzer (Hiseq 2500) based on the manufacturer’s protocol by Novogene Inc., Beijing. *De novo* assembly was done using CLC Genomics Workbench v11.0 (Qiagen Inc., Aarhus, Denmark) and Geneious R11 (Kearse et al. 2012) with reference of *K. tomentosa*. Finally, the sequence was annotated by PGA (Qu et al. 2019) and Geseq online program (Tillich et al. 2017).

Approximately 4.0 G raw data were assembled of *B. daigremontiana* chloroplast genome sequence with length 150,058 bp, which submitted to GenBank with the accession number MT954417. The cp genome was showed a typical quadripartite structure, consisted of a large single-copy region with 82,164 bp (LSC) and small single-copy region with 17,042 bp (SSC), separated by a pair of inverted repeat regions with 25,426 bp (IRs), each. The whole genome sequence GC content is 37.62%, while in LSC, SSC and IR regions are 35.63%, 31.39% and 42.92%, respectively. A total of 131 genes were predicted in the whole chloroplast genome of *B. daigremontianum*, including 86 protein-coding genes, 37 tRNA genes, and 8 rRNA genes.

The phylogenetic analysis of *B. daigremontiana* and other eight species of Crassulaceae were based on Bayesian inference using Mybayes v3.2.7 (Ronquist et al. 2012) and maximum likelihood (ML) method using RaxMLGUI v2.0 (Silvestro and Michalak 2012) with *Paeonia brownii* Dougl. ex Hook (Accession number: MH191385) as outgroup. The tree was constructed using GTRGAMMA substitution model. From the result of two phylogenetic tree, *B. daigremontianum* was close to *K. tomentosa* formed a strongly supported clade (Figure 1). These researches will provide useful information for the phylogenetic and evolutionary studies of the genus *Bryophyllum* and *Kalanchoe* in the future.

**Figure 1.**
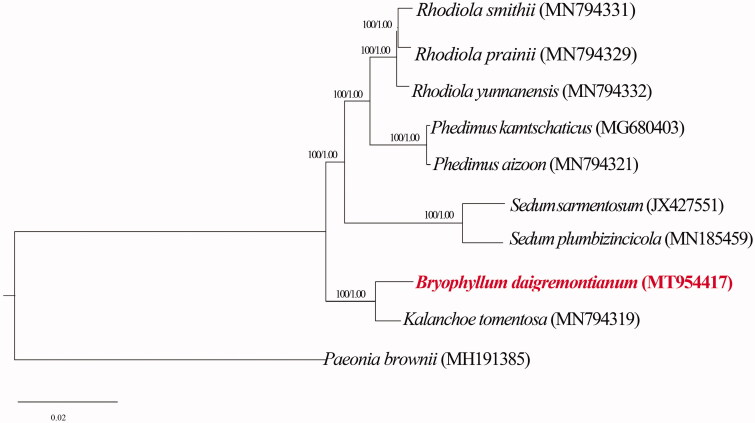
The phylogenetic tree based on 9 cp genomes of Crassulaceae family and 1 cp genomes of Paeoniaceae family as outgroup. Numbers near the branch mean the bootstrap value of RaxML and Bayesian posterior probability of MrBayes, respectively.

## Data Availability

The genome sequence data that support the findings of this study are openly available in GenBank of NCBI at https://www.ncbi.nlm.nih.gov/ under the accession no. MT954417. The associated SRA number is PRJNA668018.
